# Iron chelation and multiple sclerosis

**DOI:** 10.1042/AN20130037

**Published:** 2014-01-30

**Authors:** Kelsey J. Weigel, Sharon G. Lynch, Steven M. LeVine

**Affiliations:** *Department of Biological Sciences, University of Notre Dame, Notre Dame, IN, U.S.A.; †Department of Neurology, University of Kansas Medical Center, Kansas City, KS, U.S.A.; ‡Department of Molecular and Integrative Physiology, University of Kansas Medical Center, Kansas City, KS, U.S.A.

**Keywords:** deferasirox, deferiprone, deferoxamine, experimental autoimmune encephalomyelitis, iron deposition, Aβ, amyloid-β, AD, Alzheimer’s disease, APP, amyloid precursor protein, BBB, blood–brain barrier, CIS, clinically isolated syndrome, CNS, central nervous system, EAE, experimental autoimmune encephalomyelitis, EDSS, Expanded Disability Status Scale, FSS, functional status scale, HIF-1α, hypoxia-inducible factor 1α, ICARS, International Cooperative Ataxia Rating Scale, IL, interleukin, MBP, myelin basic protein, MFC, magnetic field correlation, MOG, myelin oligodendrocyte glycoprotein, MPTP, 1-methyl-4-phenyl-1,2,3,6-tetrapyridine, MS, multiple sclerosis, NBIA, neurodegeneration with brain iron accumulation, 6-OHDA, 6-hydroxydopamine, PD, Parkinson’s disease, PKAN, pantothenate kinase-associated neurodegeneration, PPMS, primary progressive MS, PS_1_, presenilin 1, RBC, red blood cell, RNS, reactive nitrogen species, ROS, reactive oxygen species, RRMS, relapsing remitting MS, SPMS, secondary progressive MS, SWI, susceptibility-weighted imaging, TBARS, thiobarbituric acid reactive substances, TNF-α, tumor necrosis factor α

## Abstract

Histochemical and MRI studies have demonstrated that MS (multiple sclerosis) patients have abnormal deposition of iron in both gray and white matter structures. Data is emerging indicating that this iron could partake in pathogenesis by various mechanisms, e.g., promoting the production of reactive oxygen species and enhancing the production of proinflammatory cytokines. Iron chelation therapy could be a viable strategy to block iron-related pathological events or it can confer cellular protection by stabilizing hypoxia inducible factor 1α, a transcription factor that normally responds to hypoxic conditions. Iron chelation has been shown to protect against disease progression and/or limit iron accumulation in some neurological disorders or their experimental models. Data from studies that administered a chelator to animals with experimental autoimmune encephalomyelitis, a model of MS, support the rationale for examining this treatment approach in MS. Preliminary clinical studies have been performed in MS patients using deferoxamine. Although some side effects were observed, the large majority of patients were able to tolerate the arduous administration regimen, i.e., 6–8 h of subcutaneous infusion, and all side effects resolved upon discontinuation of treatment. Importantly, these preliminary studies did not identify a disqualifying event for this experimental approach. More recently developed chelators, deferasirox and deferiprone, are more desirable for possible use in MS given their oral administration, and importantly, deferiprone can cross the blood–brain barrier. However, experiences from other conditions indicate that the potential for adverse events during chelation therapy necessitates close patient monitoring and a carefully considered administration regimen.

## Abstract 2

Abnormal iron deposition begins early in the disease course of multiple sclerosis, and this iron may facilitate pathogenesis. Iron chelation is an intervention strategy that can ameliorate iron-related pathogenic events, but it has associated risks.

## IRON LEVELS ARE ALTERED IN THE CNS OF MULTIPLE SCLEROSIS PATIENTS

Iron is an essential element that is used for basic and specialized cellular functions throughout the body and in the CNS (central nervous system). In the brain, iron is used in enzymes involved in mitochondrial respiration, neurotransmitter biosynthesis, myelination, RNA and DNA repair, cell proliferation, cellular defenses, etc. (Furukawa et al., 1992; Beard, 2001; Beard et al., 2003; Todorich et al., 2009; Yi et al., 2009; Crichton et al., 2011). To manage this iron, there are numerous proteins involved with iron transport and storage within the brain (Connor and Menzies, 1995; Crichton et al., 2011). Iron is also a key element used by proliferating inflammatory cells (Golding and Young, 1995; Kuvibidila et al., 1999, 2002; Beard 2001), which can enter the CNS during disease in MS (multiple sclerosis).

Several different MRI approaches have been used to detect iron in the CNS of MS patients: T2 hypointensity, MFCs (magnetic field correlations), R2* relaxometry, and SWI (susceptibility-weighted imaging) (recently reviewed by Bagnato et al., 2013). MFC imaging has been used to quantitate relative iron levels in deep gray matter structures of MS and control subjects (Ge et al., 2007). The MFC value was 39.5% greater in the putamen, 30.6% greater in the thalamus and 24% greater in the globus pallidus in MS patients compared to controls (Ge et al., 2007). SWI phase images have been used to quantitate iron within MS lesions, and on average, lesions had an excess of 47 μg of iron (per gram of tissue) compared to the surrounding tissue, although the surrounding tissue had slightly less iron than white matter (Haacke et al., 2009). As MRI techniques have evolved, higher field strengths have been utilized (e.g., SWI at 7T) that have greater sensitivity for iron detection over other techniques (Bagnato et al., 2011, 2013; Al-Radaideh et al., 2013). The enhanced sensitivity has been important for revealing details of iron deposition, e.g., microbleeds and the accumulation of iron around the edges of lesions (Bagnato et al., 2011, 2013), and for detecting deposition of iron early in the disease course (Al-Radaideh et al., 2013).

The accumulation of iron is thought to begin early in the disease course since multiple MRI studies have detected increased iron accumulation in gray matter structures (e.g., caudate, globus pallidus, pulvinar and/or putamen) in patients with CIS (clinically isolated syndrome) (Ceccarelli et al., 2010; Hagemeier et al., 2012; Al-Radaideh et al., 2013), which is often a prelude to the development of MS. The evidence for an effect of age on iron accumulation in CIS is mixed. In one study, no significant age-related effects on iron signals (susceptibility) were observed in a SWI study of CIS patients (22–53 years, 10 females, 9 males, no disease modifying therapy or steroid given 1 month before scan) (Al-Radaideh et al., 2013). However, age was positively correlated with iron accumulation in the caudate, putamen, and globus pallidus in a R2* relaxometry study of CIS and RRMS (relapsing remitting MS) patients (CIS: ~25–~40 years, 22 females, 10 males, 6/32 on interferon-β; RRMS: ~32–~42 years, 22 females, 15 males, 1/37 intravenous immunoglobulins, 2/27 natalizumab, 4/27 glatiramer acetate, 14/37 interferon-β) (Khalil et al., 2009). Notably, iron accumulation in the CNS of patients with CIS is not due to systemic iron overload (Ristori et al., 2011).

Besides CIS, iron deposition also has been observed by MRI in patients with RRMS (Khalil et al., 2009), SPMS (secondary progressive MS) (Ceccarelli et al., 2009), PPMS (primary progressive MS) (Bagnato et al., 2013), benign MS (Ceccarelli et al., 2009), and pediatric MS (Ceccarelli et al., 2011; Hagemeier et al., 2013). Several studies suggest that the level of iron deposition might be associated with disease duration (Bakshi et al., 2000) and/or signs of disease severity, e.g., EDSS (Expanded Disability Status Scale), brain atrophy, cognitive changes, and lesion load (Bakshi et al., 2000; Bermel et al., 2005; Tjoa et al., 2005; Brass et al., 2006; Ge et al., 2007; Zhang et al., 2007; Neema et al., 2009). In support of this notion, SPMS patients had more iron deposition than RRMS patients (Bakshi et al., 2000, 2002), which is consistent with an association of increased disease severity; however, benign MS with a longer duration of disease had comparable levels of iron deposition to that in SPMS (Ceccarelli et al., 2009) even though benign MS is milder than SPMS. This may suggest that length of disease rather than disease severity is a major contributing factor (Ceccarelli et al., 2009). Other factors that could influence iron deposition or its detection include the measurement technique (Al-Radaideh et al., 2013), the patient's age, lesion burden, lesion type [demyelination and/or axonal transection, BBB (blood–brain barrier) disruption, etc.], and/or location of lesion (LeVine et al., 2013).

During the course of MS, iron accumulates or becomes redistributed within various deep gray matter, cortical, or white matter structures. In gray matter, iron deposits have been observed in a variety of structures including the thalamus, putamen, caudate, globus pallidus, pulvinar, and Rolandic cortex (Bakshi et al., 2000, Ge et al., 2007, Ceccarelli et al., 2009; Williams et al., 2012; Al-Radaideh et al., 2013). Typically, increased iron deposition is bilaterally represented in matching deep gray matter structures across hemispheres (Bakshi et al., 2002; Khalil et al., 2009), although, in some instances, there are indications of differences in the relative levels of iron deposition between left and right structures (Bermel et al., 2005; Ceccarelli et al., 2009, 2010, 2011). Iron deposits in white matter or associated with cortical lesions do not necessarily have matching bilateral representations as the deposits are often associated with inflammatory lesions, i.e., at the rim of active plaques or in a perivascular location (Bagnato et al., 2011; Williams et al., 2012; Mehta et al., 2013).

Histochemical localization of iron deposits has been correlated with images obtained by high field strength MRI in both EAE (experimental autoimmune encephalomyelitis) (Williams et al., 2011) and MS (Pitt et al., 2010; Bagnato et al., 2011) specimens. In these studies, histological staining for iron revealed iron deposits in microglia and macrophages at the rim of cortical and white matter (both chronic and active) lesions (Hammond et al., 2008; Pitt et al., 2010; Bagnato et al., 2011). In addition, staining was observed around vessels within macrophages, microglia and in the surrounding white or gray matter (Bagnato et al., 2011; Williams et al., 2011). The staining around vessels was putatively due to micro-hemorrhaging (Bagnato et al., 2011; Williams et al., 2011). Staining was also observed in oligodendrocytes and myelin, which was present in control subjects, and some subpial staining of iron was observed in a variety of cells including neurons, but this staining was suggested to be an artifact (Bagnato et al., 2011).

In other histochemical studies, pathological iron deposits in MS have been observed in punctate structures within neurons suggestive of damaged mitochondria or lipofuscin (LeVine, 1997). In white matter of MS or animal subjects with EAE, iron deposits have been observed in macrophages, reactive microglia, extravasated RBCs (red blood cells) (hemosiderin), granular deposits, astrocytes, and/or damaged axons (Craelius et al., 1982; Adams, 1988, 1989; LeVine, 1997; Forge et al., 1998; Pedchenko and LeVine, 1998; Williams et al., 2011; Hametner et al., 2013). Thus, iron undergoes redistribution as cells and/or structures, e.g., myelin, get damaged or the uptake of iron can be altered when cells experience stress, e.g., during inflammation. Indeed, the concentration of iron in white matter plaques was found to be less than in normal-appearing white matter (Hametner et al., 2013). Iron in normal-appearing white matter is found mainly in myelin and oligodendrocytes, but the concentration of iron was found to decrease in chronic progressive MS compared to white matter from controls, and the decrease was correlated with the duration of disease (Hametner et al., 2013). In normal subjects, the iron content increases with age in both deep and subcortical white matter, but this age-related increase in iron content was not observed in MS (Hametner et al., 2013).

There are a number of proteins involved in the management of iron during disease. For example, in white matter lesions (i.e., initial and active lesions), as well as in periplaque white matter, a microarray study revealed that the messages for the iron storage protein ferritin (i.e., mitochondrial ferritin and heavy and light ferritin) are up-regulated while the expression of the gene for export of iron from mitochondria, frataxin, is down-regulated in initial lesions (Hametner et al., 2013). Immunohistochemical localization of ferritin revealed ferritin-positive oligodendrocytes in normal-appearing white matter (Bagnato et al., 2011), but the oligodendrocytes decreased in concentration in the periplaque region of patients with active MS with a corresponding increase in ferritin-positive microglia (Hametner et al., 2013). At the rim of acute lesions, iron was present in ferritin-positive microglia/macrophages (Bagnato et al., 2011; Hametner et al., 2013), although there were also ferritin-positive cells that were not enriched with iron (Hametner et al., 2013).

In addition to storage, iron import is also regulated by changes in factors such as the transferrin receptor. Microarray studies found that the expression of the mRNA for the transferrin receptor is up-regulated in normal-appearing white matter in MS patients (Graumann et al., 2003), in periplaque white matter, and possibly in active lesions (Hametner et al., 2013), but these studies were focused on gene expression and did not study transferrin protein expression. Immunohistochemistry and radioactively-labeled holo transferrin studies revealed an up-regulated expression of the transferrin receptor in the periplaque region in MS brain tissue (Hulet et al., 1999). The cells expressing the transferrin receptor in the periplaque region appeared to be oligodendrocytes, while the expression of the transferrin receptor in normal-appearing white matter from MS brain tissue was limited to vessels similar to that seen in normal controls (Hulet et al., 1999). Other proteins involved with iron import/transport or that act as a ferrireductase or ferroxidase were also identified to have an up-regulated expression by microarray studies in periplaque white matter (Solute carrier family 11, member 2), in initial lesions (scavenger receptor class A, member 5, this is also present in active lesions), or in both periplaque and initial lesions (solute carrier family 39, member 14; transient receptor potential cation channel, subfamily C, member 6; STEAP family member 3; hephaestin–this last protein is also elevated in active lesions) (Hametner et al., 2013), but caution should be exercised since, except for hephaestin, the expressions of these proteins were not studied. Hephaestin, which facilitates with the efflux of intracellular iron (Schulz et al., 2011), was observed in oligodendrocytes and astrocytes (Hametner et al., 2013). There were lower numbers of hephaestin-labeled cells in the normal-appearing white matter in MS patients compared to white matter in controls (Hametner et al., 2013). Within the periplaque white matter, the levels of hephaestin expression within individual oligodendrocytes was elevated suggesting that the cells were actively exporting iron, while more astrocytes expressed hephaestin in active lesions (Hametner et al., 2013). A variety of additional proteins are involved with the transport and management of iron in the brain, and these proteins play a role in the accumulation and/or redistribution of iron in MS. However, since there have been recent articles that have covered the uptake of iron in the brain relative to MS, the reader is referred to these papers (e.g., Williams et al., 2012; Hare et al., 2013).

## PLAUSIBLE MECHANISMS OF IRON-INDUCED PATHOLOGY IN MULTIPLE SCLEROSIS

**Figure 1:**
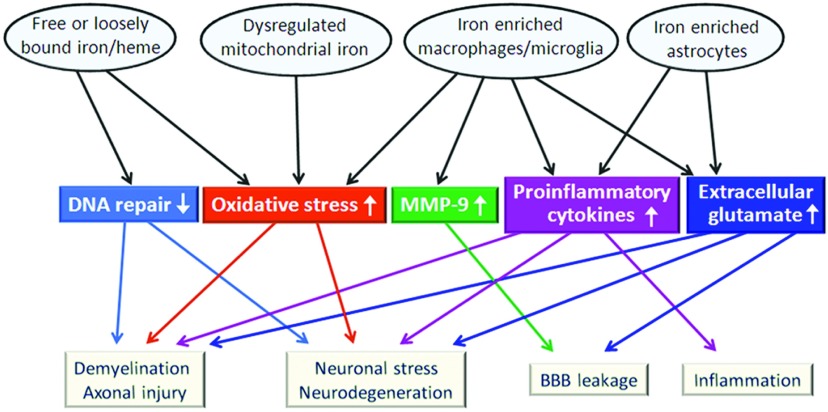
Abnormal iron deposits that are found in MS likely contribute to pathogenesis by multiple interrelated mechanisms Many of these mechanisms could be relevant for the progressive nature of disease and may be impacted by chelation therapy.

Given the range of iron deposits in MS, there are a number of possible sources for the deposited iron, which can putatively partake in various pathogenic mechanisms (Figure 1). For example, pathological iron deposits could arise from damaged structures (e.g., oligodendrocytes, myelin, endothelial cells, neurons, axons) releasing iron-containing proteins, some of which contain heme. Iron deposits observed around the plaque edge would support these cellular sources (Craelius et al., 1982; Hametner et al., 2013; Mehta et al., 2013). Additionally, perivascular inflammation, which can be a predominant feature in MS and EAE, is thought to cause damage to vessel walls leading to permeability changes including the extravasation of RBCs into the CNS and perivascular iron deposits (Adams, 1988; Wakefield et al., 1994; Forge et al., 1998; Bagnato et al., 2011; Williams et al., 2011).

Although not specific to MS, the extravasation of RBCs into the CNS is a potential instigator of tissue damage. Extravasation of RBCs would likely occur during an acute exacerbation in RRMS, when there is disruption to the BBB, and EAE studies indicate that management of breakdown products from RBCs are important contributors of the disease process (discussed below). A RBC is packed with hemoglobin and each molecule of hemoglobin contains four molecules of heme, which is an iron protoporphyrin. Upon degradation of RBCs, hemoglobin exists in two forms, either as a tetramer or a heterodimer. The predominant form is the heterodimer and reactions involving these dimers can result in tissue damaging activities (Schaer et al., 2013). To counter this, upon release into the extracellular fluid, haptoglobin binds hemoglobin, and the complex is taken up by macrophages/microglia via the CD163 receptor (Fabriek et al., 2005; Zhang et al., 2011). Of note, haptoglobin-deficient mice have more severe EAE disease than control mice with haptoglobin, thereby indicating a protective role for haptoglobin (Galicia et al., 2009). Reactions involving the remaining hemoglobin dimers can result in the release of hemin (the oxidized form of heme) (Schaer et al., 2013) and/or breakdown of hemoglobin releases heme (Robinson et al., 2009).

In addition to being concentrated in and released from RBCs, heme is used by numerous proteins in oligodendrocytes and neurons. During an inflammatory attack, e.g., an exacerbation in RRMS, or during ongoing pathology in progressive forms of MS, these cells can undergo degeneration resulting in the release of heme-containing proteins, which can contribute to pathological iron deposits. Heme, hemin, and iron can catalyze reactions leading to ROS (reactive oxygen species) and RNS (reactive nitrogen species) that can damage CNS tissue (Bian et al., 2003; LeVine and Chakrabarty, 2004; Robinson et al., 2009; Lu et al., 2011) (Figure 1). Hemopexin binds free hemin and the complex is taken up by the low-density lipoprotein receptor-related protein/CD91 receptor on macrophages (Hvidberg et al., 2005) and possibly by astrocytes and neurons, although the binding of heme to albumin may limit this uptake (Robinson et al., 2009). A recent EAE study showed that hemopexin-deficient mice developed more severe disease, i.e., earlier onset and greater disease activity, than EAE mice with hemopexin (Rolla et al., 2013). Although hemopexin binds and helps remove free heme, it is possible that hemopexin-deficient oligodendrocytes in the knockout mice were more sensitive to inflammation and/or allowed for a greater response by Th17 cells (Rolla et al., 2013), which promote pathology in EAE and MS (Jadidi-Niaragh and Mirshafiey, 2011).

Other putative uptake mechanisms for hemin are the heme carrier protein 1 or direct interaction with the lipid bilayer and possible endocytosis (Robinson et al., 2009). Iron is released from heme by heme oxygenase 1, and put into intracellular storage as ferritin (LeVine and Chakrabarty, 2004, Robinson et al. 2009). This mechanism is thought to limit the pro-oxidant effects of iron and heme, and limit disease activity, i.e., in EAE (Liu et al., 2001; Chora et al., 2007). However, if too much iron is released via heme oxygenase 1, the iron may not be properly stored and result in iron-induced oxidative stress (Robinson et al., 2009), which could worsen disease (Chakrabarty et al., 2003; Stahnke et al., 2007). Furthermore, iron deposition in microglia or macrophages is thought to enhance a pro-inflammatory environment. For instance, excess iron in an activated microglial cell line promoted the inflammatory response by these cells, e.g., increased matrix metalloproteinase-9 expression (Mairuae et al., 2011) and elevated the release of proinflammatory cytokines TNF-α (tumor necrosis factor α) and IL-1β (interleukin-1β) (Zhang et al., 2006).

Iron has been detected in pro-inflammatory macrophages and microglia present around the plaque edges, and these were more commonly observed in active RRMS compared to SPMS (Mehta et al., 2013). These iron-enriched cells did not have evidence of phagocytosing myelin debris, and were positively stained for inducible nitric oxide synthase, an activation marker for M1 polarization (Mehta et al., 2013). In culture experiments, macrophage iron uptake increased the release of TNF-α and promoted elevated production of ROS, while the presence of myelin suppressed iron uptake and the release of these molecules (Mehta et al., 2013). The authors suggest that iron-ladened M1 cells promote a chronic pro-inflammatory state at the edge of active plaques (Mehta et al., 2013). In related studies, iron induced elevated levels of pro-inflammatory cytokines, ROS, and RNS production by cultured microglial cells exposed to hypoxic conditions, and the media from these cells was found to activate caspase-3 expression, a marker of apoptosis, in primary oligodendrocytes (Rathnasamy et al., 2011) indicating that a related mechanism could occur in MS.

Iron, together with hydrogen peroxide, reduced the uptake of glutamate by astrocytes (Fernandes et al., 2011), and elevated levels of pro-inflammatory cytokines and ROS released by microglia can also lower uptake. The lowered uptake can result in higher extracellular levels of glutamate, which can promote excitotoxic damage (Domercq et al., 2007; Matute, 2011) including neuronal swelling (Rossi et al., 2013) (Figure 1). Deferoxamine administration decreased elevated glutamate levels and protected CA1 neurons in the hippocampus following hypoxia/ischaemic insult in neonatal rats (Papazisis et al., 2008). Besides potentially being toxic to neurons, glutamate could promote injury directly to oligodendrocytes, e.g., via AMPA/kainate and/or NMDA receptors (Benarroch, 2009; Matute, 2011), or potentially promote leakage of the BBB (Germanò et al, 2007; Wu et al., 2013) (Figure 1). In animal models of MS, microglia express high levels of glutaminase (Werner et al., 2001). Coupled with the finding that iron promotes increased brain glutamate levels (McGahan et al., 2005), it is possible iron stimulates glutamate-mediated cytotoxicity via microglia glutaminase in MS patients (Bolton and Paul, 2006; Domercq et al., 2007; Yu et al., 2009). In fact, the glutaminase inhibitor, 6-diazo-5-oxo-L-norleucine, was found to reduce glutamate release from LPS (lipopolysaccharide)-activated microglia *in vitro* and it delayed the onset of EAE clinical signs (Shijie et al., 2009). Furthermore, antagonists of the AMPA/kainate receptors resulted in attenuation of EAE (Pitt et al., 2000; Smith et al., 2000). It is unclear how well findings in animal studies will translate to humans as studies indicate that the expression or response of glutamate receptors in human oligodendrocytes may be substantially different (lower expression levels and resistant to excitotoxicity) from those in animals (Wosik et al., 2004; Benarroch, 2009).

Mitochondrial dysfunction may promote pathology during both acute inflammatory events as well as during the progression of neurodegeneration (Su et al., 2013). TNF-α increases the uptake of iron in microglia and astrocytes *in vitro* (Rathore et al., 2012) and TNF-α and IL-1β induce the uptake of iron by mitochondria in cultured astrocytes (Mehindate et al., 2001). Iron accumulation can induce mitochondrial dysfunction, resulting in oxidative stress (Figure 1), which has been implicated in EAE and MS by a large variety of studies (Bakshi et al., 1998; Calabrese et al., 2003; Chakrabarty et al., 2003; LeVine and Chakrabarty, 2004; Zhang et al., 2005; Mahad et al., 2008; Zhang et al., 2009; Deng et al., 2010; Mao and Reddy, 2010; Onyango et al., 2010; Srinivasan et al., 2010; Choi et al., 2011; Pelizzoni et al., 2011). Decreased mitochondrial membrane potential due to neuronal iron uptake has been shown to enhance oxidative damage (Zhang et al., 2009), and a feedback loop may occur since mitochondrial oxidative damage can lead to increased mitochondrial iron uptake (Mastroberardino et al., 2009). Of note, the iron chelator deferoxamine was shown to reduce mitochondrial oxidative stress in the penumbra of a model of transient cerebral ischemia (Im et al., 2012). And other classes of chelators (e.g., 2-pyridylcarboxaldehyde isonicotinoyl hydrazine) have been shown to mobilize mitochondrial iron better than deferoxamine (Richardson et al., 2001).

Iron may also be able to catalyze oxidative tissue injury independent of mitochondria, and this oxidative damage could amplify demyelination, promote axonal injury, and enhance neuronal stress (Figure 1). Indeed, some white matter lesions showed co-localization of iron deposits and oxidized phospholipids, and there was a weak, but significant, correlation between the iron staining and staining for oxidized phospholipids (Hametner et al., 2013). Also, DNA damage in MS, resulting from oxidative stress (Vladimirova et al., 1998), may not be repaired properly due to iron adversely affecting the DNA repair machinery (Li et al., 2009) (Figure 1).

Although various mechanisms can be theoretically attributed to iron worsening the pathological course in MS (Figure 1), the actual role of iron on the disease course is still unclear. To gain insights about the significance of iron in MS pathogenesis, results from pharmacological studies directed at controlling the toxic effects of iron, e.g., iron-catalyzed oxidative damage, or studies limiting iron deposition or promoting its removal can be examined. Here we will focus our attention on iron-chelating agents since this intervention strategy focuses on an early step in catalytic oxidative tissue damage as well as other iron-mediated pathological change, and it has potential therapeutic value for MS.

## CHELATION THERAPY

An iron chelator binds iron and promotes its removal, and in some cases a chelator has been shown to alter the distribution of iron within cells (Kakhlon et al., 2008, 2010; Sohn et al., 2008; Szuber et al., 2008). These actions can limit oxidative damage in both *in vitro* and *in vivo* settings (Wu et al., 1993; Britton et al., 2002; Eybl et al., 2002; Rajesh et al., 2004; Fisher and Naughton, 2005; Glickstein et al., 2006).

For clinical use, chelators are designed to either promote clearance of excess iron by excretion or maintain safe iron levels in situations that pose a risk for developing toxic levels of iron, e.g., following multiple blood transfusions for β-thalassaemia (Porter, 2009; Brittenham 2011). Three chelators approved for therapeutic use are deferoxamine (a.k.a., desferrioxamine, desferoxamine), deferasirox, and deferiprone, and sometimes they are used in combination with one another (Mitchell et al., 2007; Cappellini et al., 2009; Porter, 2009; Brittenham, 2011; Kwiatowski, 2011; Fernandes, 2012). Deferoxamine (MW 656.8) is a hexadentate ligand that binds iron in a 1:1 molar ratio while the tridentate ligand deferasirox (MW 373.4) binds iron in a 2:1 molar ratio, and deferiprone (MW 139.2) interacts with iron in a bidentate fashion forming a 3:1 molar complex with iron. Deferoxamine is administered by intravenous injection or subcutaneously with iron complexes being cleared mainly by biliary and urinary routes, while deferasirox and deferiprone are taken orally with the former typically eliminated by the hepatobiliary route and the latter via the kidney (Brittenham, 2011; Kwiatkowski, 2011). Although iron chelators have been shown to effectively reduce systemic iron levels, only deferiprone was shown to effectively cross the normal BBB (Fredenburg et al., 1996; Habgood et al., 1999; Ma et al., 2012).

## IRON CHELATION THERAPY HAS YIELDED MIXED RESULTS FOR NON-DEMYELINATING CNS DISEASES WITH IRON ACCUMULATION

A large variety of diseases result in iron accumulation in the CNS, and in numerous instances, iron chelation has been explored as a possible mechanism to ameliorate disease activity. In many of these diseases, iron accumulates in similar areas of the brain as in MS, or iron has a putative pathogenic mechanism that is shared between the disease and MS. Thus, examining the use of chelators for these conditions could have relevance for designing studies for MS.

Friedreich's ataxia, which is due to GAA repeats in the frataxin gene (Rajeswari, 2012), results in dysregulation of mitochondrial iron metabolism and enhanced oxidative stress catalyzed by iron (Vaubel and Isaya, 2013). In MS, mitochondria can undergo oxidative damage (Lu et al., 2000), and frataxin expression is down-regulated in initial MS lesions further indicating mitochondrial injury (Hametner et al., 2013). In addition, iron accumulates in the dentate in both MS (Tjoa et al., 2005) and in Friedreich's ataxia (Koeppen, 2011), although there are other affected structures that are not shared between the diseases.

Friedreich's ataxia has been the target of iron chelator studies. A 6-month clinical trial (10–15 mg of deferiprone/kg, twice daily) resulted in reduced iron accumulation in the dentate as detected by MRI (R2* relaxation), improved scores on the ICARS (International Cooperative Ataxia Rating Scale) and lessening of some clinical signs, e.g., gait, balance (Boddaert et al., 2007). In a second clinical study, combined therapy of deferiprone (10 mg/kg, twice daily) with idebenone (20 mg/kg per day) [an analog of coenzyme Q_10_, with antioxidant properties, that protects the heart and possibly the CNS in Friedreich's ataxia patients (Mariotti et al., 2003; Parkinson et al., 2013)] was evaluated in 20 Friedreich's ataxia patients (Velasco-Sánchez et al., 2011). After 11 months of treatment there was less iron detected in the dentate nucleus as assessed by MRI (T2*), and steady ICARS scores resulted from improved kinetic function, but worsening posture and gait compared to baseline in Friedreich's ataxia patients (Velasco-Sánchez et al., 2011).

NBIA (neurodegeneration with brain iron accumulation) diseases [e.g., aceruloplasminemia, neuroferittinopathy, and PKAN (pantothenate kinase-associated neurodegeneration) 2] result in iron accumulation in the globus pallidus together with an additional gray matter structure(s) (Schneider et al., 2013). For aceruloplasminemia and neuroferittinopathy, additional structures include the putamen, caudate and possibly the thalamus (Schneider et al., 2013). Interestingly, these structures are the same deep gray matter structures that display iron accumulation in MS (Bakshi et al., 2000, Ge et al., 2007, Ceccarelli et al., 2009; Williams et al., 2012; Al-Radaideh et al., 2013). Iron chelation therapy has been explored for NBIA diseases.

Deferoxamine, which does not cross the intact BBB, did not have value for PKAN when it was administered for 6 months at 250 mg/day by intramuscular injection (Gallyas and Környey, 1968). However, deferiprone, which does cross the BBB, did lessen brain iron but did not improve clinical symptoms in a study of nine patients with PKAN (12.5 mg of deferiprone/kg, twice daily for 6 months) (Zorzi et al., 2011). Another deferiprone study (15 mg/kg, twice daily) observed modest clinical improvement in two out of three patients with PKAN (Abbruzzese et al., 2011), while a case study of combined oral deferiprone with intrathecal baclofen reported some modest improvements compared to intrathecal baclofen alone (Pratini et al., 2013). Two patients with neuroferittinopathy were given deferoxamine (4 g/week) for up to 14 months and a third patient was given deferiprone (2 g, three times a day) for 2 months; no benefits were observed in two of these patients and a third worsened (Chinnery et al., 2007). In three patients with aceruloplasminemia, deferasirox (start dose of 17–20 mg/kg per day; the dose was reduced or stopped at different times for each patient with treatment lasting 1 week to 5 months) was found to improve liver iron accumulation but did not lessen iron accumulation in the brain (Finkenstedt et al., 2010), and studies testing deferoxamine found no improvement in brain iron accumulation (Loréal et al., 2002; Mariani et al., 2004). However, a case study that examined the combination of plasma (as a source of ceruloplasmin) together with deferoxamine reported an improvement in clinical signs, e.g., less ataxia and reduced choreoathetosis (Miyajima et al., 1997), but it was unclear which compound was potentially responsible for the observed effect. Deferiprone treatment (15 mg/kg, twice daily) resulted in an improvement in clinical signs (e.g., gait, dysarthria, dystonia) and lessening of brain iron in two patients with NBIA of undetermined cause (Forni et al., 2008; Kwiatkowski et al., 2012).

A rare CNS condition, superficial siderosis, is caused by repeated subarachnoid hemorrhage (Fearnley et al., 1995; Levy, et al., 2007; Yamawaki 2012) that results in the deposition of hemosiderin in the subpial layer of the brain and/or spinal cord, and/or in cranial nerves (Yamawaki 2012). Hemosiderin/iron is deposited around vessels in MS (Adams, 1988) and EAE (Williams et al., 2011) subjects, and numerous studies indicate that the management of heme is relevant for the disease course in EAE (see the Plausible mechanisms of iron-induced pathology in MS section above). Thus, the outcome of chelation studies on superficial siderosis could have particular relevance for the perivascular iron deposits observed in MS.

Due to the accumulated iron within hemosiderin, iron chelation therapy has been tested for superficial siderosis. Decreased hemosiderin, as detected by lessening of the T2 hypointensity, was observed in four of nine subjects following a 90-day course of deferiprone (15 mg/kg, twice daily), while three subjects had no change and two patients had slightly more hemosiderin (Levy and Llinas, 2012a). Patient reporting of symptoms indicate that four patients had an improvement in symptoms, four were unchanged, and two were worse (Levy and Llinas, 2012a). Longer term administration of deferiprone, i.e., 38 months, in one patient revealed resolution of ataxia and hearing loss, which were present prior to the initiation of treatment (Levy and Llinas, 2012b).

In addition to the rare disorders discussed above, the much more common neurodegenerative disorders PD (Parkinson's disease) and AD (Alzheimer's disease) also have elevated levels of CNS iron deposits. Iron-catalyzed oxidative stress has been implicated as a contributing factor in the pathogenesis of PD (Weinreb et al., 2013) and AD (Hare et al., 2013) as well as MS (LeVine and Chakrabarty, 2004; Williams et al., 2012; Hametner et al., 2013; Mehta et al., 2013). Besides oxidative stress, other pathological features, such as activated microglia and progressive neurodegeneration, have been noted to be shared between MS, PD, and AD (Amor et al., 2010; Lassmann, 2011).

PD patients have increased iron concentrations in dopamine neurons within the substantia nigra pars compacta (Weinreb et al., 2013), which is a primary site of pathology in this disease. Furthermore, iron has been co-localized with α-synuclein in Lewy bodies and iron may facilitate α-synuclein aggregation (Castellani et al., 2000; Weinreb et al., 2013). Due to the elevated levels of iron in the substantia nigra together with the presence of iron within the Lewy bodies and the role of iron in oxidative stress, iron chelators have been examined in experimental settings for this disease. Intracerebroventricular delivery of deferoxamine prior to 6-OHDA (6-hydroxydopamine) administration, which causes nigrostriatal dopaminergic neuron degeneration, resulted in multiple measures (e.g., striatal dopamine levels, tyrosine hydroxylase activity and dopaminergic-related behavioral responses) indicating less disease activity compared to vehicle (Ben-Shachar, et al., 1991, 1992; Youdim et al., 2004). In addition to intracerebroventricular administration, deferoxamine has been given via the intranasal route as this delivery has been shown to permit the entry of molecules into the brain that might not otherwise cross the intact BBB (Hanson et al., 2009). Intranasal administration of deferoxamine was associated with improvement in motor function, but this was incomplete, and it did not prevent dopaminergic neuron cell death in a PD model that involved a midbrain injection of an adeno-associated viral vector encoding for α-synuclein (Febbraro et al., 2013).

Besides deferoxamine, delivery of deferasirox to the substantia nigra zone compacta resulted in less neuronal loss and preservation of striatal dopamine levels compared to vehicle in rats given 6-OHDA (Dexter et al., 2011). This effect was also observed with systemic administration of deferoxamine, deferasirox, or deferiprone with the latter giving the strongest effect (Dexter et al., 2011). Orally delivered deferiprone was also shown to lessen the loss of tyrosine hydroxylase cells in the substantia nigra zone in mice exposed to the Parkinson’s-inducing agent MPTP (1-methyl-4-phenyl-1,2,3,6-tetrapyridine); this protection was approximately twice that offered by intraperitoneal administration of deferoxamine (Devos et al., 2013). Deferiprone administration also resulted in lowered labile iron pools in mitochondria, reduced measures of oxidative stress, reduced depletion of dopamine, and improved motor function in the mice (Devos et al., 2013). Studies were also performed in humans. Patients with early PD were given deferiprone (15 mg/kg, twice daily) in a randomized, placebo-controlled, double-blind clinical trial (Devos et al., 2013). Nineteen patients were selected to begin treatment at the start of the study while eighteen additional patients were given placebo for six months, and then began administration of deferiprone. Those patients receiving early administration of deferiprone had significantly better outcome on the Unified Parkinson's Disease Rating Scale and improved R2* MRI measures of iron deposits in the substantia nigra than the delayed group of patients at 6 and 12 months (Devos et al., 2013). After the initial 12 month pilot study, patients were re-randomized and selected to terminate treatment at either 18 or 24 months. Those with treatment extended to 24 months had improved measures of motor function compared to those ending at 18 months (Devos et al., 2013). Two cases of neutropenia (at months 3 and 7) and one case of agranulocytosis (at month 3) were identified from blood counts performed on a weekly basis. These conditions resolved by 2 weeks following the withdrawal of deferiprone (Devos et al., 2013).

Additional chelators including clioquinol, M30, and the green tea component epigallocatechin-3-gallate have also revealed protective properties in various models of PD (Weinreb et al., 2013). Additionally, overexpression of the heavy chain of ferritin driven by the rat tyrosine hydroxylase promoter, producing ferroxidase activity that converts ferrous iron into ferric iron, attenuated the reduction of striatal dopamine and lowered the loss of substantia nigral dopaminergic neuronal cells in transgenic mice exposed to MPTP compared to control mice exposed to MPTP (Kaur et al., 2003). However, overexpression of ferritin also resulted in degeneration of these cells that was associated with aging (Kaur et al., 2007), and the degeneration was likely related to an increase in the labile pool of iron (Kaur et al., 2009).

Iron accumulation has been detected in both plaques and tangles in AD (LeVine, 1997; Smith et al., 1997) and has been shown to facilitate oxidative stress (Smith et al., 1997; Sayre et al., 2000; Huang et al., 2004; Castellani et al., 2012), which is prevalent in both sporadic and familial AD and may help drive disease progression (Hunter et al., 2013). Evidence indicates that metals, such as iron, are able to interact directly with Aβ (amyloid-β) peptide, a principle component of the β-amyloid lesions present in AD (Huang et al., 2004). When redox-active iron is reduced in the presence of Aβ, production of hydrogen peroxide and hydroxyl radical can occur and could be responsible for oxidative damages in AD patients (Huang et al., 2004). Additionally, brain iron dysregulation has been associated with plaque formation, e.g., enhanced iron accumulation was present at the early disease stage including the initiation of plaque formation in the cortex of an APP (amyloid precursor protein)/PS_1_ mouse model (Leskovjan et al., 2011). However, iron was not significantly associated with plaques at the later stages (56 weeks) when normalized to protein density and assessed by Thioflavin S-staining of APP/PS_1_ mouse brain tissue followed by X-ray fluorescence microscopy (Leskovjan et al., 2011). Together, these data provide a rationale to examine iron chelation therapy in AD or its animal models (Atwood et al., 2003; Reznichenko et al., 2006).

Intranasal delivery of deferoxamine significantly reduced memory loss in APP/PS_1_ mice (Hanson et al., 2012; Guo et al., 2013a), induction of phosphorylated tau that contributes to tangle formation was suppressed (Guo et al., 2013b), Aβ burden was reduced, and amyloidogenic APP processing was inhibited (Guo et al., 2013a). In addition, when treated with intranasal delivery of deferoxamine, transgenic mice that experience an accumulation of hyperphosphorylated tau showed significant improvement in radial arm water maze performance, which is a measure of spatial memory function (Fine et al., 2012). Finally, when AD patients were administered intramuscular injection of deferoxamine (125 mg, twice daily; 5 days a week for 2 years), there was a significant reduction in the rate of decline of daily living skills as compared to a placebo group (Crapper McLachlan et al., 1991).

The mixture of outcomes for chelation therapy for the diseases described above could be due to a variety of factors including: dosage, administration route and/or duration of treatment, timing of treatment relative to the disease course, the underlying pathogenic mechanisms of the disease being studied, the properties of chelator used (e.g., ability to penetrate the BBB), whether a peripheral effect from chelation therapy could influence disease in the CNS, etc. Furthermore, most of the studies had a very small sample size, which could lead to highly unreliable results, e.g., there could be missed positive effects or alternatively that observed benefits may not be applicable to a larger sample size.

Like the diseases described above, it is possible that there could be a mixture of outcomes for MS following the administration of a chelator. Since there are different types of MS (e.g., RRMS, PPMS, SPMS) that involve different pathogenic mechanisms, it is possible that one form may be more responsive to chelation treatment than another, or that the treatment regimen will need to be catered to the specific type of MS.

## IRON CHELATION IN EAE

Chelation therapy has been examined in EAE models of MS. In 1984, Bowern et al. administered 70 mg/day of deferoxamine via osmotic pump for 7 days to 120–150 g Lewis/JC rats given guinea pig spinal cord homogenate as an encephalitogen. Deferoxamine prevented the development of symptoms when it was administered prior to the onset of clinical signs, and it hastened the recovery process when it was given after clinical evidence of disease started (Bowern et al., 1984; Table 1). In contrast, deferoxamine at a similar dosage did not suppress EAE when it was administered during the preclinical stage to rats given a purified encephalitogen, i.e., MBP (myelin basic protein) (Willenborg et al., 1988; Table 1). Thus, deferoxamine likely acted to impede the exposure of encephalitogenic proteins in guinea pig spinal cord homogenates when it was administered preclinically, and this limited the immune response, but this was not the case when purified MBP was administered in the preclinical stage (Willenborg et al., 1988). Also deferoxamine did not lessen disease when it was given during the preclinical stage of EAE in rats given passive transfer of cells from MBP or spinal cord homogenate-injected rats (Willenborg et al., 1988; Table 1). However, deferoxamine treatment started 1 day before transfer of cells and lasted a total of only 7 days (Willenborg et al., 1988). Unfortunately, the onset day of clinical disease was not listed for the passive transfer experiments (Willenborg et al., 1988), but it can take as long as 5–7 days after cell transfer for animals to display clinical signs (Sedgwick et al., 1987). Deferoxamine (40, 80 or 157 mg/kg, three times a day) did ameliorate disease activity when it was administered during active disease in male SJL mice given purified MBP and pertussis toxin injections (Pedchenko and LeVine, 1998; Table 1). In a guinea pig (strain 13) optic neuritis EAE model, deferoxamine conjugated to hydroxyethyl starch, which increases its plasma half-life, lessened BBB leakage, and may have reduced pathology, but the latter difference did not reach significance (Guy et al., 1994). However, this conjugated compound did not lessen disease in SJL mice given MBP, which may be due to it having an ~100-fold increase in size compared to deferoxamine (Pedchenko and LeVine, 1998) (Table 1).

**Table 1:** Iron chelation in EAE models MBP, myelin basic protein; MOG, myelin oligodendrocyte glycoprotein; PLP, proteolipid protein.

Species	Strain	Encephalitogen	Drug	Dosage	Timing of chelator administration	Outcome	Reference
Rat (female)	Lewis/JC	Guinea pig spinal cord homogenate	Deferoxamine	70 mg/day implanted s.c. pump	Prior to onset of clinical signs	Reduced clinical and pathological signs	Bowern et al., 1984
Rat (female)	Lewis/JC	Guinea pig spinal cord homogenate	Deferoxamine	70 mg/day implanted s.c. pump	Overlapping with clinical signs	Blocked development of clinical signs during treatment	Bowern et al., 1984
Rat (female)	Lewis/JC	Guinea pig spinal cord homogenate	Deferoxamine	70 mg/day implanted s.c. pump	Started after the onset of clinical signs	Hastened recovery and reduced pathological signs	Bowern et al., 1984
Rat (female)	Lewis (RT-1^1^)	MBP from guinea pig	Deferoxamine	70 mg/day implanted s.c. pump	Prior to onset of clinical signs	No effect (similar to vehicle-treated animals)	Willenborg et al., 1988
Rat (female)	Lewis (RT-1^1^)	Passive transfer of cells from MBP immunized or spinal cord homogenate injected rats	Deferoxamine	70 mg/day implanted s.c. pump	Started prior to onset of clinical signs, unclear if treatment overlapped with presentation of clinical signs	No effect (similar to vehicle-treated animals)	Willenborg et al., 1988
Mice (male)	SJL/J	MBP	Deferoxamine	40–~160 mg/kg; three times a day	During active disease	Lessened clinical signs and reduced some pathology	Pedchenko and LeVine, 1998
Guinea pig	Strain 13	Guinea pig spinal cord homogenate	HES-conjugated deferoxamine	100 mg/kg per day	Starting at the time of encephalitogen injection	Lessened BBB leakage and possible small effect on pathology	Guy et al., 1994
Mice (male)	SJL/J	MBP	HES-conjugated deferoxamine	0.7–2.8 g/kg once per day	During active disease	No clear effect	Pedchenko and LeVine, 1998
Mice (female)	SJL/J	PLP_139–151_	Deferiprone	150 mg/kg (~3 mg/mouse); twice daily via gavage	During active disease	Suppressed clinical signs and reduced some pathology	Mitchell et al., 2007
Rat (female)	Lewis	MBP	Dexrazoxane	5 mg/kg; 3 i.v. injections	Prior to and at onset of clinical signs	Sample size too small; possible lessening of clinical signs	Weilbach et al., 2004
Rat (female)	Lewis	Adoptive transfer	Dexrazoxane	25 mg/kg; 3 i.v. injections	Overlapping with active disease	Lessened clinical signs and pathology	Weilbach et al., 2004
Mice (female)	SJL/J	PLP_139–151_	Apoferritin	750 μg, twice daily	During active disease	Suppressed clinical signs	LeVine et al., 2002
Mice (female)	C57BL/6	MOG_35–55_	Clioquinol [copper and zinc chelator, but it can affect brain iron content and it may also chelate iron (Lei et al., 2012); see Iron chelation in EAE section]	30 mg/kg per day via gavage	From injection of encephalitogen to end of study	Suppressed clinical signs and pathology	Choi et al., 2013

The observations that deferoxamine blocked active disease and expedited recovery in the Lewis/JC rat model (Bowern et al., 1984; Table 1) and it reduced disease in SJL mice given MBP (Pedchenko and LeVine, 1998) suggest that chelation can affect ongoing disease. This notion is supported by a study on deferiprone (150 mg/kg, twice daily) which was shown to suppress disease activity when it was administered after the onset of clinic signs in female SJL mice given an encephalitogenic PLP peptide and pertussis toxin injections (Mitchell et al., 2007; Table 1). Also, in Lewis rats given adoptive transfer of MBP T-cells, treatment with the iron chelator dexrazoxane (25 mg/kg given on days 1, 3 and 5 after the transfer of cells) led to reduced clinical signs and fewer T-cells and macrophages entering the spinal cord compared to PBS administration (Weilbach et al., 2004; Table 1). In rats with active disease induced by immunization with MBP, dexrazoxane administration (5 mg/kg) at days 8, 10 and 12 post-immunization (disease onset was ~11 days) led to reduced clinical signs, but the reduction was not statistically significant, and treatment did not influence the proliferation of T-cells collected from lymph nodes (Weilbach et al., 2004).

Apoferritin can bind large amounts of iron and protect against oxidative injury (discussed in LeVine et al., 2002). When it was administered during active disease (750 μg, twice daily), apoferritin lessened clinical signs of EAE in SJL female mice given PLP peptide as an encephalitogen and pertussis toxin injections (LeVine et al., 2002) (Table 1).

Clioquinol is a chelator of copper and zinc, but it may also interact with iron (Lei et al., 2012). Clioquinol administration can lower serum iron levels (Schäfer et al., 2007), and it can reduce brain iron levels in normal mice and in mouse models of PD (Yassin et al., 2000; Kaur et al., 2003; Lei et al., 2012), although the reverse was observed in models of AD (Schäfer et al., 2007; Grossi et al., 2009). Clioquinol was tested in EAE mice, though the authors were more focused on the zinc and copper chelation properties (Choi et al., 2013). Clioquinol at 30 mg/kg per day was administered to C57BL/6 mice via gavage starting at the day of encephalitogen injection of a peptide of myelin oligodendrocyte glycoprotein (MOG_35–55_), or starting after the onset of clinical signs, and continued until the time of sacrifice (Choi et al., 2013). Significant reductions of clinical and pathological signs were observed for both administration regimens (Choi et al., 2013; Table 1).

The results from four chelators (deferoxamine, deferiprone, dexrazoxane, clioquinol) indicate that EAE disease can respond to this form of therapy. However, these studies are limited in scope, e.g., they have been of relatively short duration, and important questions remain. For example, it is unknown whether chelation therapy will benefit chronic progressive forms of disease or whether it will reduce the relapse rate in relapsing remitting disease. Furthermore, more detailed measures could answer outstanding questions, e.g., whether chelation therapy results in less abnormal iron deposits and/or less oxidative stress, reduces the number of mitochondrial alterations that occur during disease, and/or whether it has effects on other pathological mechanisms relevant to MS. Putative therapeutic mechanisms of chelation therapy for MS are discussed below.

## THERAPEUTIC MECHANISMS

Studies examining therapeutic mechanisms indicate that iron chelation therapy could benefit MS by several different avenues, e.g., regulating the immune response, limiting iron-catalyzed oxidative damage, redistributing iron within cells, and inducing cellular defense mechanisms (Table 2).

**Table 2:** Putative therapeutic mechanisms of chelators in MS

Pathological target	Putative mechanism	Examples of supporting references	Form of MS most likely to benefit
T- and B-cells	Limit cell proliferation in the periphery and possibly in the CNS	Lederman et al., 1984	RRMS
		Warnke et al., 2009	
		Sweeney et al., 2011	
Cytokines	Modulate cytokine production in the periphery and possibly in the CNS	Del Vecchio et al., 2002	RRMS
		Weibach et al., 2004	
		Leung et al., 2005	
Perivascular iron (heme) deposits	Limit iron catalyzed tissue damage to myelin, the BBB, etc.	Wu et al., 1993	RRMS
		Guy et al., 1994	
		Nakamura et al., 2004	
	Stabilize HIF-1α	Schofield and Ratcliffe, 2005	
		Semenza, 2012	
		Singh et al., 2012	
Demyelination and axonal injury leading to release of iron (or heme) containing products	Limit amplification of tissue damage by iron catalyzed reactions	Wu et al., 1993	RRMS/SPMS
		Kadiiska et al., 1995	
		Nakamura et al., 2004	
		Merkofer et al., 2006	
	Stabilize HIF-1α	Schofield and Ratcliffe, 2005	
		Semenza, 2012	
		Singh et al., 2012	
Activated and iron enriched macrophage, microglia or astrocytes	Limit production of ROS	Rajesh et al., 2004	RRMS/SPMS
	Limit production of proinflammatory cytokines	Molina-Holgado et al., 2008	
		Rathnasamy et al., 2011, 2013	
	Limit production of MMP-9	Mairuae et al., 2011	
	Improve glutamate uptake to lower extracellular levels	Fernandes et al., 2011	
Altered mitochondrial function in neurons	Limit ROS production by mitochondria	Richardson et al, 2001	RRMS/SPMS
		Liang et al., 2008	
		Im et al., 2012	
	Limit caspase-3 activation by mitochondria	Zhang et al., 2009	
Enhanced iron deposition in deep gray matter	Limit glutamate excitotoxicity	Papazisis et al., 2008	RRMS/SPMS
		Yu et al., 2009	
	Protect against oxidative stress	Molina-Holgado et al., 2008	
	Reduce brain iron uptake	Crowe and Morgan, 1994	
	Lessen iron induced inhibition of DNA repair machinery	Li et al., 2009	
	Stabilize HIF-1α	Schofield and Ratcliffe, 2005	
		Semenza, 2012	
		Singh et al., 2012	

A chelator can directly influence the immune response. For example, deferoxamine has been shown to limit the proliferation of T- and B-cells (Lederman et al., 1984) and it reduced cytokine production by peripheral blood mononuclear cells (Leung et al., 2005) *in vitro*. A more recent study examined the effects of deferiprone on peripheral blood T-cells collected from control and MS patients (Sweeney et al., 2011); the proliferation of both CD4^+^ and CD8^+^ T-cells stimulated by anti-human CD3 and anti-human CD28 antibodies was suppressed in the presence of 75 μM deferiprone compared to vehicle (Sweeney et al., 2011). Furthermore, T-cells collected from rats immunized with MBP and then treated *in vitro* with dexrazoxane (5 μM) displayed a minimal reduction in proliferation; however, the addition of iron enhanced the proliferation of these cells, and this enhanced response could be blunted with dexrazoxane, indicating that iron acted as a co-stimulator for proliferation (Weilbach et al., 2004). Inhibition of proliferative responses by a chelator is likely due to sequestering iron from ribonucleotide reductase, which is used for DNA synthesis (Cooper et al., 1996; Kuvibidila et al., 2002). The effects of deferiprone on the immune response have also been observed in thalassemia major patients where it lowered the increased levels of CD8^+^ cells, IL-2, and TNF-α (Del Vecchio et al., 2002). Other drugs used for MS also target the immune response; of note, teriflunomide, a disease-modifying therapy for MS (He et al., 2012), limits the proliferative responses of B- and T-cells by targeting DNA synthesis, i.e., inhibiting dihydroorotate dehydrogenase (Warnke et al., 2009). It would be useful to determine if deferiprone also acted on the proliferative response by B-cells given the role of B-cells in MS pathogenesis (Lehmann-Horn et al., 2013). Other studies also suggest a connection between disease activity, iron, and the immune response. For instance, iron-deficient male B10.PL mice failed to develop EAE while mice with normal levels of iron did develop EAE following MBP and pertussis toxin injections (Grant et al., 2003).

The oxidative activity in leukocytes from MS patients is higher than for control subjects (Ferretti et al., 2006), and chelation therapy can affect the oxidative potential of cells in the blood. For example, deferoxamine and deferiprone reduced cytotoxic aldehydes in plasma (an indicator of oxidative stress), and increased glutathione peroxidase activity in RBCs in β-thalassemia patients compared to non-chelated patients (Bartfay et al., 1999). Deferasirox caused a reduction of oxidative markers, e.g., ROS and lipid peroxidation in RBCs (and increased glutathione), in patients with myelodysplastic syndromes receiving blood transfusions of RBCs (Ghoti et al., 2010). In another study, monocytes from patients with Eales’ disease were shown to have elevated iron levels compared to control subjects (Rajesh et al., 2004). These cells produced higher levels of hydroxyl radical and TBARS (thiobarbituric acid reactive substances) when stimulated with phorbol-12-myristate acetate compared to healthy controls, and two chelators (deferoxamine or diethylenetriaminepentacetic acid) reduced hydroxyl radical formation and TBARS production by these cells (Rajesh et al., 2004). Thus, chelation therapy could act on peripheral monocytes to limit the oxidative potential of these cells (Table 2).

Iron chelators could be acting within the CNS of EAE and MS subjects to limit oxidative tissue damage by preventing redox cycling (Merkofer et al., 2006) or limiting the formation of hydroxyl radical (Kadiiska et al., 1995) (Table 2). Some iron chelators could gain limited access to the CNS due to the breakdown of the BBB that occurs in MS and EAE (Pedchenko and LeVine, 1998), while other chelators, such as deferiprone, can cross the BBB (Fredenburg et al., 1996; Habgood et al., 1999; Ma et al., 2012) and act in the CNS independent of BBB leakage. Once in the CNS, the chelator could bind iron and impede its availability to partake in reactions leading to ROS and RNS that can cause tissue damage (Bian et al., 2003; LeVine and Chakrabarty, 2004; Lu et al., 2011). In support of this, deferoxamine reduced a marker of oxidative damage (8-hydroxyl-2′-deoxyguanosine) in the brains of rats given a model of intracerebral hemorrhage (Nakamura et al., 2004). This result suggests that chelation therapy could have a protective effect against micro-hemorrhages that are observed in MS (Adams, 1988; Bagnato et al., 2011). Chelation therapy could also protect against iron released from degenerating cells due to disease activity (Figure 1). In support of this, deferiprone protected neurons *in vitro* from iron-induced toxicity (Molina-Holgado et al., 2008). Additionally, deferoxamine limited several measures of lipid peroxidation in a model of experimental uveitis in Lewis rats (Wu et al., 1993). Since a recent study found evidence suggesting iron-induced oxidative damage to myelin in MS (Hametner et al., 2013), the findings from Wu et al. (1993) indicate that chelation could ameliorate this putative pathogenic mechanism in MS.

Iron-enriched microglia have been shown to have an elevated production of pro-inflammatory cytokines and ROS production (Zhang et al., 2006; Rathnasamy et al., 2011; Mehta et al., 2013), which could promote inflammation in MS. Deferoxamine has been shown to reduce the release of these pro-inflammatory molecules (Rathnasamy et al., 2011) (Table 2), although the mechanism may be via the induction/stabilization of HIF-1α (hypoxia-inducible factor 1α) (Rathnasamy et al., 2013) (discussed below).

Iron chelation therapy could limit the uptake of iron in the brain (Crowe and Morgan, 1994) and limit mitochondria dysfunction (Table 2). Iron uptake can increase in mitochondria following oxidative damage (Mastroberardino et al., 2009). In a kainite-induced status epilepticus rat model, chelatable iron levels increased in mitochondria and chelation therapy [*N,N*'-bis (2-hydroxybenzyl) ethylenediamine-*N,N*'-diacetic acid] lessened mitochondrial changes and improved neuronal survival (Liang et al., 2008). Deferiprone can also cause the redistribution of iron within cells, e.g., shift labile iron from mitochondria (Sohn et al., 2008). Furthermore, since oxidative damage to DNA has been observed in MS (Vladimirova et al., 1998), it is relevant to note that iron can inhibit DNA repair machinery in neurons and in brain extracts *in vitro*, and the inhibition was lessened in the latter by deferoxamine (Li et al., 2009). Removal of iron from the brain may require repeated or extended administration of a chelator since very short-term chelation therapy (less than 3 h) to young rats (15 days) did not appreciably reduce radiolabel iron in the brain that was administered 2 or 24 h prior to chelation (Crowe and Morgan, 1994).

Iron chelation could also lead to protection of CNS structures via a preconditioning mechanism. HIF-1α is a transcription factor that responds to hypoxic conditions by inducing the expression of an array of proteins for adaptive responses to a lower oxygen state (Singh et al., 2012). In normoxia conditions, HIF-1α becomes hydroxylated, e.g., by prolyl hydroxylase, which is iron-dependent and utilizes oxygen as a substrate, resulting in proteasomal degradation of HIF-1α (Semenza, 2012). In hypoxic conditions, the lower oxygen level limits hydroxylation and, as a result, HIF-1α stability and activity are increased (Semenza, 2012). Iron chelators are thought to remove iron from hydroxylases, thereby inhibiting their activity and limiting proteosomal degradation of HIF-1α, allowing it to be active (Semenza, 2012). Thus, deferoxamine is thought to act by stabilizing the expression of HIF-1α (Jiang et al., 1997; Schofield and Ratcliffe, 2005), and in a study of the rat kidney, an analog of deferiprone, CP94, also stabilized HIF-1α (Baek et al., 2011). Preconditioning with the iron chelator deferoxamine provided protection against CNS injury, e.g., in ischemia models (Prass et al., 2002; Zhu et al., 2008; Singh et al., 2012), and increased the viability of oligodendrocytes following exposure to TNF-α *in vitro* (Yao et al., 2008).

Some of the downstream genes regulated by HIF-1α include proteins involved with iron metabolism, e.g., transferrin, transferrin receptor, and heme oxygenase (Mole, 2010; Weinreb et al., 2013). Hence, the induction/stabilization of HIF-1α could perform a preconditioning role in MS; in fact, HIF-1α and downstream genes that it controls, such as transferrin receptor, are induced in normal-appearing white matter from MS patients (Graumann et al., 2003). This induction may help oligodendrocytes perform their normal function while under duress in MS, and other HIF-1α up-regulated genes may provide neuroprotection (Graumann et al., 2003). Methylprednisolone, which is commonly used to treat disease relapses, induced HIF-1α in cultured oligodendrocytes and protected them from excitotoxic cell death, i.e., exposure to AMPA (Sun et al., 2010). Low-dose prophylactic administration of deferoxamine (40 mg/kg per day) or deferasirox (10 or 20 mg/kg per day) reduced the volume of injury in a stroke model in mice, but their mechanism of action was thought to be independent of HIF-1α (Zhao and Rempe, 2011).

Iron chelation could impact the disease course in MS by a number of mechanisms (Table 2). Systemic effects of chelation, e.g., on the peripheral immune system, could be relevant since autoimmune reactions are generally thought to have a predominant pathogenic role in MS. Although this is important, other compounds currently in use also limit the immune response. Other mechanisms such as reducing oxidative tissue damage (e.g., via blockage of the formation of reactive intermediates or induction of HIF-1α) could be very relevant in helping to limit damage that is progressing slowly even in the absence of overt inflammation. Information about these mechanisms can be used to facilitate the design of iron chelation clinical trials in order to optimize the potential for therapeutic impact.

## CHELATION IN MULTIPLE SCLEROSIS

An initial trial of deferoxamine administration was conducted on 12 MS patients as defined by the clinical criteria described by Weiner and Ellison (1983) and by a positive MRI scan (Norstrand and Craelius, 1989). No patient was enrolled that received an immunosuppressant within 3 months of deferoxamine administration (Norstrand and Craelius, 1989). Nine patients received 20 mg/kg per day and three patients received ~30 mg/kg per day (or 2 grams/day) for 5 days/week for 3 months. Using EDSS and/or FSS (Functional Status Scale) as outcome measures, two to four patients improved (two with marked improvement), three had slight improvement, four to six had no improvement, and one was slightly worse (Norstrand and Craelius, 1989; Table 3).

**Table 3:** Chelation studies in MS patients

Study	Number of study subjects	Dosing	Unable to complete the study	Measure	Improved	Slight improvement	No change	Worsened	Adverse events
Norstrand and Craelius, 1989	12 MS	9 patients on deferoxamine 20 mg/kg per day; 3 patients on ~30 mg/kg per day; 5 days/week for 3 months	0/12	EDSS	2/12	3/12	6/12	1/12	3/12 urinary tract infection
				FSS	4/12	3/12	4/12	1/12^1^	
Lynch et al., 1996	9 PPMS; 10 SPMS	1-week course of deferoxamine 2 g/day followed by a second week of 1 g/day	1/19	EDSS	9/18 (3 months); 9/18 (6 months); 5/18 (12 months)		7/18 (3 months); 6/18 (6 months); 7/18 (12 months)	2/18 (3 months); 3/18 (6 months); 6/18 (12 months)	1/19 nausea, widespread local reaction, mild hearing loss, blurred vision^2^; 2/19 fever, nausea, muscle aches^3^; 2/19 mild fatigue and anorexia; 1/19 urinary tract infection; 1/19 worsened visual evoked response; 19/19 localized redness at infusion site
				FSS	13/18 (3 months); 10/18 (6 months); 7/18 (12 months)		5/18 (3 months); 8/18 (6 months); 7/18 (12 months)	0/18 (3 months); 1/18 (6 months); 4/18 (12 months)	
Lynch et al., 2000	5 PPMS; 4 SPMS	1-week course of deferoxamine; 2 g/day followed by a second week of 1 g/day; repeated at 3-month intervals	8 courses of treatment (6/9 patients)^4^; 7 courses of treatment (1/9 patients); 5 courses of treatment (2/9 patients)	EDSS	1/9 (0.5 points)		3/9	5/9 (0.5 points)	1/9 abdominal pain^5^; 1/9 nausea and lethargy^4^; 9/9 localized redness at infusion site
				Other	1/9 weakness and numbness of hands resolved; 1/9 weakness of left leg resolved; 1/9 improvement of ataxia lasting ~2 months after course of treatment				

^1^Not the same patient for EDSS and FSS.

^2^Treatment was discontinued and all symptoms resolved shortly thereafter.

^3^Occurred during the winter and thought to be due to a virus.

^4^Due to lethargy and nausea, one patient had 5 days of 2 g/day followed by 5 days of 1 g/day.

^5^Not thought to be treatment related as it occurred many weeks after the sixth treatment.

A second deferoxamine trial examined 19 patients with MS as defined by the criteria by Poser et al. (1983) together with an abnormal MRI and a reduction of the EDSS of ≥0.5 over the previous 2 years (Lynch et al., 1996). Patients with heart disease, low iron, low ferritin, or chronic anemia were not enrolled (Lynch et al., 1996). Out of the 19 patients, 9 had PPMS and 10 had SPMS. Patients were given a 1-week course of 2 g/day followed by a second week of 1 g/day without any other immunosuppressant for at least 3 months after treatment (Lynch et al., 1996). One patient was unable to complete the course of treatment due to side effects that resolved following discontinuation of the drug administration. The remaining 18 patients did not have a decline in their neurological status during the two weeks of treatment or shortly following treatment. At 3 months following drug administration, 16 patients had some improvement or remained unchanged in their EDSS while two patients had declined. With more time after the completion of treatment, the EDSS for 15 out of 18 patients improved or remained unchanged while three patients declined at 6 months, and the EDSS for 12 out of 18 patients remained improved or unchanged with six worsening at 12 months (Lynch et al., 1996; Table 3).

A third deferoxamine study was conducted on nine patients with MS as defined by Poser et al. (1983), together with an abnormal MRI and a ≥ 0.5 reduction for the EDSS during the prior 2 years (Lynch et al., 2000). Four patients had SPMS and five had PPMS. Patients on an immunosuppressant or corticosteroid within the previous 6 months or that had low iron, low ferritin, heart disease or chronic anemia were not enrolled (Lynch et al., 1996). Patients were given 2 g/day for 7 days followed by 1 g/day for 7 days and this 2-week course was repeated every 3 months. Two patients completed five courses of treatment, one completed seven courses, and six completed eight courses of treatment across 2 years. One patient had an improvement of 0.5 in their EDSS, three patients were unchanged, and five patients worsened by 0.5 points. Some patients reported improvements not covered by the EDSS, e.g., resolving of numbness and weakness in the hands, improvement of leg weakness, and a lessening of ataxia (Lynch et al., 2000; Table 3).

Despite being limited to testing only deferoxamine, the three small pilot clinical trials leave open the possibility that iron chelation could be a beneficial therapeutic approach. It is important to note that the disease course typically progresses over time, and although treatment would ideally result in improvement or stop progression, a positive intervention could still be obtained if disease progression occurred at a slower rate in treated patients than for control MS subjects. Unfortunately, these small studies did not have control MS subjects, nor were the studies blinded.

Multiple factors may explain why there have not been more clinical trials that examined the therapeutic value of chelators in MS since 2000. First was the need to determine whether iron contributes to pathology or whether it is an epiphenomenon of ongoing pathology. Recent studies have established that iron deposition begins early in the disease course (Al-Radaideh et al., 2013) and can promote pathology, e.g., oxidative damage and enhanced production of pro-inflammatory cytokines (Mehta et al., 2013; Hametner et al., 2013). Second was the need for the development, and acceptance in clinical practice, of iron chelators that avoided some of the problems associated with deferoxamine. The development of newer chelators (e.g., deferasirox and deferiprone) offers the advantage of having a greatly improved administration route, i.e., oral administration versus 6–8 h of subcutaneous infusion for deferoxamine. Third was the need for a chelator to cross the BBB. Deferiprone crosses the BBB (Fredenburg et al., 1996; Habgood et al., 1999; Ma et al., 2012), and thus, it would have a better opportunity to act on pathogenesis throughout the CNS rather than relying on an abnormal BBB for access.

Given the ability to penetrate the BBB, deferiprone could act in the CNS by multiple mechanisms. Deferiprone could access and limit the function of immune cells that infiltrated the CNS, similar to that observed *in vitro* (Sweeney et al., 2011). It could bind perivascular iron deposits or capture iron released from degenerating oligodendrocytes/myelin (Williams et al., 2012; Hametner et al., 2013), and thereby limit the potential spreading of oxidative stress. It could lessen the enhanced production of pro-inflammatory cytokines by iron-enriched macrophages/microglia (Zhang et al., 2006; Mehta et al., 2013) by limiting iron uptake by these cells. It could possibly induce HIF-1α expression (Baek et al., 2011), resulting in the induction of a protective stress response pathway. And it could redistribute iron within cells, e.g., remove labile iron in mitochondria (Sohn et al., 2008), which could facilitate the management of iron within cells such as those in deep gray matter in MS. In the future, access of chelators to the brain will likely improve as recent studies have investigated ways to increase their permeability into the brain (Roy et al., 2010; Ma et al., 2012; Liddell et al., 2013).

Additional clinical trials testing chelation therapy in MS patients are likely forthcoming. Identifying the correct dosage, timing, and type of MS that would be the most probable to benefit from this form of therapy remains to be determined. It is possible that chelation therapy could lessen disease progression in SPMS, e.g., via targeting the iron accumulation in deep gray matter structures, however, an extended study may be required to demonstrate a therapeutic effect. It is also possible that chelation therapy could influence the disease course in RRMS by helping to regulate the immune response, or limiting iron-catalyzed oxidative stress.

Future studies should include a measurement of iron levels as assessed by MRI, as this would be directly related to one of the objectives of chelation therapy, i.e., reduce abnormally accumulated iron, and it could be a more sensitive outcome measure than a scoring system based on clinical signs. In addition, other MRI measures of disease activity should be included in the assessment of drug efficacy.

## SAFETY OF IRON CHELATION THERAPY

The three pilot studies on deferoxamine in MS subjects revealed that generally patients tolerated the treatment, despite an arduous delivery system, e.g., subcutaneous infusion over 6–8 h multiple times per week. Out of forty patients among the three studies (Norstrand and Craelius, 1989; Lynch et al., 1996, 2000), four developed urinary tract infections (Norstrand and Craelius 1989; Lynch et al., 1996); two patients had muscle aches, fever and nausea, but was possibly due to a viral infection (Lynch et al., 1996); one patient experienced abdominal pain, but this appeared to be unrelated to treatment since it occurred many weeks following his last administration (Lynch et al., 2000); two patients had anorexia and mild fatigue (Lynch et al., 1996); and one patient developed lethargy and nausea (Lynch et al., 2000) (Table 3). In two of the studies, localized redness occurred around the injection site in all 28 patients, and in one of these patients the reaction around the injection was extensive (Lynch et al., 1996, 2000; Table 3). This patient also had nausea, mild hearing loss and blurred vision, which all resolved following discontinuation of treatment (Lynch et al., 1996).

Despite the willingness of study patients to tolerate the arduous dosing regimen during a relatively short study, it is unlikely that the average patient could tolerate this regimen for prolonged periods of time, which could be a limiting factor for the use of deferoxamine in MS. Other chelators, such as deferasirox and deferiprone, are delivered orally and thus avoid a major drawback with deferoxamine. Since deferiprone crosses the BBB (Fredenburg et al., 1996; Habgood et al., 1999; Ma et al., 2012), it makes a desirable candidate for future testing in MS.

Anemia in MS patients is an important consideration before beginning treatment with an iron chelator. In one study, 28 out of 72 MS patients had iron-deficient anemia (Rodrigo et al., 2011), while another study found that although MS patients were more likely to have iron-deficient anemia than controls, only 24 out of 898 MS patients were anemic (Kang et al., 2010). Furthermore, a subgroup of patients that presented with clinical signs consistent with MS displayed low values for iron parameters and remained stable after iron supplementation (van Toorn et al., 2010; van Rensburg et al., 2012). Even in patients that are not anemic, identifying the correct dose of a chelator would require a careful balance, i.e., keeping the dosage low enough to prevent anemia and other complications while allowing a high enough dosage to impact iron deposits in the brain. Thus, the relatively high dosages of chelators used in acute EAE studies will need to be adjusted to levels that are safe for prolonged administration in clinical trials involving MS patients. Although a large number of studies indicate a protective role of iron chelation against oxidative stress, one *in vitro* study found that a low concentration of deferiprone could promote oxidative stress (Cragg et al., 1998). It appears, however, that results from *in vivo* studies have not supported this finding thus far.

Iron chelation therapy requires close monitoring of patients for side effects. Although many of the common side effects can be reversed with discontinuation of treatment, or by reducing the dosage levels, some side effects are life threating and require immediate attention. Of particular importance, neutropenia, agranulocytosis, and thrombocytopenia have been reported with deferiprone use; thus, monitoring of blood counts is advised (FERRIPROX; Jamuar and Lai, 2012). Other deferiprone side effects include various forms of gastrointestinal disturbance (e.g., nausea, abdominal discomfort, vomiting, diarrhea), headache, joint disorder, back pain, increased or decreased appetite, etc. (FERRIPROX; Jamuar and Lai, 2012). Patients on deferasirox have also experienced neutropenia, agranulocytosis and thrombocytopenia requiring monitoring of blood cells (Cappellini and Pattoneri, 2009; EXJADE). Gastrointestinal disturbance (e.g., abdominal pain, diarrhea, nausea, and vomiting), skin rash, hepatic failure, gastrointestinal hemorrhage, acute renal failure, hearing loss, vision abnormalities, etc., have also been observed in patients receiving deferasirox (Cappellini and Pattoneri, 2009; EXJADE). Finally, side effects associated with deferoxamine include a variety of visual and auditory disturbances, injection site reactions, gastrointestinal disturbances (diarrhea, vomiting, nausea, abdominal discomfort), renal failure, dizziness, neuropathy, etc. (Desferal).

## CONCLUSIONS

While many medications have been found to limit the exacerbation rates in relapsing MS, disability in patients with MS continues to accumulate over time, highlighting the need for better therapeutics. The growing body of evidence establishing that abnormal iron deposits accumulate in MS, and that this iron could contribute to pathogenesis, indicate that iron chelation may be a new way to ameliorate disease progression. While iron chelation could yield benefits, it poses many challenges in this patient population.

Continuation of studies that examine iron chelation as a possible therapy for MS is supported by multiple pieces of evidence: (i) abnormal iron deposits are present in MS brains and their accumulation begins early in the disease course; (ii) iron is known to facilitate pathogenic mechanisms, such as oxidative stress, that are thought to be relevant for MS pathogenesis; thus, chelation could affect an aspect of the disease that at present has not been impacted by other disease modifying agents; (iii) chelation ameliorates active EAE and improves outcomes in other models of CNS diseases suggesting it could ameliorate MS; (iv) monitoring of iron deposits in the CNS can be performed by MRI; and (v) chelation therapy has become easier to administer, i.e., oral delivery, and newer chelators that cross the BBB could have greater impact on the disease course.

Factors that favor a cautionary approach to chelation therapy for MS include: (i) as of yet, there is limited evidence indicating a prominent role of abnormal iron deposits in MS pathogenesis; (ii) chelation therapy can have side effects, some of which can be severe and require close monitoring; (iii) patients with MS do not have systemic iron overload; thus, the dose used in these patients would likely need to be lower than that needed for standard therapy; (iv) combination of a drug for iron chelation with a standard disease-modifying drug currently used for MS could increase the risk of adverse events, e.g., greater risk of infection; and (v) it is not clear which type of MS would most likely benefit from chelation therapy, when administration should begin, or for how long.

In conclusion, we support further studies that examine whether iron chelation therapy can ameliorate the disease course in MS patients. The possibility that iron chelation therapy could reduce oxidative stress and thus impact the more degenerative aspects of MS is very appealing. MS is a very heterogeneous disease; therefore multiple studies on varying MS populations might be needed to identify the appropriate group of patients that would benefit from this type of therapy. However, these studies should be carried out only after careful planning of the study design as well as having the physicians and patients cautiously weighing the risks and benefits.
